# Gleanings from Our Exchanges

**Published:** 1873-02-01

**Authors:** 


### Gleanings from Our Exchanges.

### THE ABORTIVE TREATMENT OF SMALL-POX.

BY. W. E. BESSEY, M.D. MONTREAL.

In view of the loathsome character of this disease, its excessively contagious nature, its mortality in persons unprotected by vaccination, the hideous deformity and disfigurement which is a frequent result of its attacks on the one hand, and, on the other, the many evidences of its entire dependence upon *a particular disease germ*, engendered, preserved and multiplied under certain favorable conditions, as shown by the success with which it can be destroyed, prevented and controlled by attention to habits of cleanliness, ventilation, and especially its destructibility by disinfectants, there seems to be not a shadow of doubt, so far as experience can guide us, that this *entity*, be it emanation, germ or fungus, is incapable of resisting the destructive action of certain chemical agents, when brought into direct contact with it. Considering the constitutional disturbance, fever, and eruption, which characterize this disease, evidences of the presence and operation of this poison in the human system, which must have been introduced in infinitesimal quantity by the lungs, or by the stomach, and which must therefore have been multiplied an indefinite number of times in the system, according to the degree of susceptibility of the patient, or the suitability of the soil in which it was thus transplanted, by being taken into the system; moreover, as it is evident that nature treats this as it does every other morbid poison, by at once making efforts to cast it off as an enemy, or alienate it, I determined to try what success would follow an attempt to destroy the poison in the blood, and prevent the continuation of its ravages in the system, by bringing in direct contact with it, in the circulation, substances which, when used as disinfectants, had not only succeeded in destroying its contagiousness, but also in eradicating the disease itself.

I have proceeded upon the hypothesis that if the disease depends upon a distinct *germ, or primal cause*—which it is self-evident that it must—and that this *entity* or *disease germ* is capable of multiplication or reproduction in the system to an indefinite extent; and that the symptoms of small-pox are the resulting constitutional disturbance from its presence and reproduction, and the eruption the casting out or effort of nature to rid itself of this enemy; and that this eruption and other emanations from the patient are contagious—then, the shortest route to a successful eradication of such a poisonous influence from a community, neighborhood or family, must be to attack, neutralize, and destroy the *disease germ* itself in each individual patient, by the use of such agents as are likely to be successful in accomplishing the feat. In this way the ravages of the disease would be cut short in the subject of its attack; nature would be prevented from sinking exhausted from its effects; its power of contagion destroyed and its ravages confined to the limits of a few isolated cases; and, by a wise use of disinfectants in suspected localities, the fountain source destroyed from whence new cases might spring up and so extend the ravages of this dire disease. To meet these indications, I selected carbolic acid as a disinfectant and antiseptic agent capable of destroying the *disease germ*, whatever may be its nature, whether an exhalation, a fungoid, an atmospheric influence, or a *disease germ* capable of being taken into the system by injestion, absorption, or inhalation.

If atmospheric germs, when introduced into open wounds, are capable of occasioning suppuration, setting up putrefaction and preventing the healing of wounds, as maintained by Dr. Lemaire, of Paris, and Mr.

Lister, and carbolic acid is capable of destroying these germs and preventing suppuration, thus promoting the healing of wounds, as it has been amply proven to be capable of doing; if it has been successful as an antiseptic and disinfectant in multitudes of ways, in destroying disease emanations, and in opposing the spread of this and other contagious diseases—scarlatina, measles, typhus and typhoid, cholera included—must it not be through the power which it possesses of destroying the *disease germs*, in whatever form, of whatever nature, and under whatever circumstances, brought into contact with them, even in the sanguineous fluid itself, as in pyæmia in the human subject? Its suitability therefore as an agent calculated to destroy the small-pox virus in the system cannot be questioned, and especially when used in solution with glycerine, an agent which possesses such power of penetrability as to find its way to the most minute cells of the bony structures themselves. I therefore selected carbolate of glycerine as the remedy best calculated to perform the work required of any remedy administered with such an object in view. The more perfectly to attain to the required *desideratum*, namely, a thorough antiseptic and disinfectant remedy, I combined the sulphite of soda in the prescription, as being a remedy well calculated, by virtue of the sulphurous acid which it contains, to destroy any parasites or vegetable fungus which might at any time be present, or have any lot or part in producing or shaping the course of an attack of the disease, and which are assumed to exist in various forms of putrid fevers and other forms of germinal disease. Moreover, sulphite of soda is a remedy of acknowledged value in many forms of disease depending upon a blood poison. The sulphurous acid which is evolved when the salt comes in contact with the acids of the stomach I suppose to act as an antiseptic and disinfectant, while the soda, forming other combinations, may act as a simple alkali or an aperient. For internal administration, then, I devised the following mixture:

℞. Acid, carbolic, ʒ j; sodæ sulphitis, ʒ x; aquæ ad. ℥ vj. Of this a dose proportionate to age of patient. For infants, one-half a teaspoonful; for children up to about seven, one teaspoonful; and for adults, a dessert-spoonful (or four teaspoonsful) every third hour, to be administered as early as possible in the disease.

When there was much fever, and little action of the skin and kidneys, I gave the following as a diaphoretic every hour, until the feverish or congestive stage of the disease had passed over.

℞. Potass. chloras, ʒ ij; liquor ammon. acet. ℥ ij; spts. eth. nit., ℥ ij; aquæ ad., ℥ vj. A teaspoonful for a child every hour; four times as much for an adult.

In cases where the eruption had already appeared before beginning the treatment, a topical application was required, which would at once destroy the contagious emanation from the vesicle or pustules, and at the same time remove the distressing itching present in most cases. To meet this indication, I prescribed a carbolate of glycerine, as follows:

℞. Acid carbolici, ʒ ij; glycerine, ℥ iij. To be applied to the face and other portions of the body on which the eruption had already appeared, once or twice a day with a feather.

I was not long waiting for an opportunity for putting this plan into practice. Mr. A., residing in Wolfe street, called upon me to visit his wife, upon a Saturday evening in April last. I found her suffering from symptoms premonitory of small-pox, high fever, great thirst, pains in limbs and back, tongue furred, etc.

Prescribed pulv. Doverii, grs. x, every six hours, with drinks of hot gruel *ad libitum*. Saw her again on Monday. The characteristic eruption of small-pox had now made its appearance copiously over the neck and face, arms and chest, and upon the inside of thighs—high fever and great thirst. Patient was nursing a child eight months old, and was the mother of a family of five other small children. I recommended immediate isolation of children, or removal of mother to hospital. Neither recommendation would be acceded to. I then prescribed the acid carbolici and sulphite of soda mixture, a dessert-spoonful dose every third hour, and gave the diaphoretic mixture at intervals of every two hours, with simple gruel or milk diet. A gentle laxative being required by the costive habit of body, I ordered a Seidlitz powder once or twice a day, as might be necessary to preserve a lax condition of the bowels. No external application was made use of in this case. On the second day the fever had abated, the pustules had begun to decline, and by the sixth day after the appearance of the eruption it had entirely disappeared, and the patient felt well enough to sit up, but was not permitted, for prudential reasons. The child continued to nurse throughout without manifesting the slightest illness after the first two or three days. None of the family contracted the disease. The patient exhibited no trace or mark of the disease after recovery. This gentleman afterwards informed me that he gave several bottles of the prescription to French Canadians in the neighborhood, members of whose families were suffering from the disease, in all of whom its action was alike satisfactory. Indeed, I had sufficient proof of this in the number of persons of that nationality who afterwards applied to me for "that particular prescription."

Case II.—Mrs. R.'s child, Murray street: This child was aged two years. When seen, the eruption was in full bloom, but distinct and copious. The child had been tried three several times with vaccination during its infancy, but each time without success. The face and head were considerably swollen, the skin very red, the child restless, and manifesting considerable internal distress by moans and cries, etc. The bowels had been costive. Ordered a moderate dose of castor oil. Pulse 140; pupils contracted; breathing regular but frequent ; kidneys acting as usual. Fearing congestion of the brain, I added to the febrifuge mixture, usually prescribed, tr. aconit. rad. gtt., ss. doses, to be given every hour until the fever abated. I ordered the carbolate of glycerine (diluted) to be applied over the whole surface with a feather, wherever the eruption existed, twice a day. The carbolate of soda mixture (or carbolic acid and sulphite of soda) I gave every three hours in doses of 1 gr. carbolic acid to 10 grs. soda sulphis, and recommended milk diet only, with an occasional mild dose of castor oil if necessary. On the second day following the pulse had fallen to 96, the pustules had begun to pit and wither, the feverish condition was entirely gone, and by the seventh day the pustular eruption had withered away to a dry scurf or scale, and was rapidly falling off, and without leaving a solitary trace of their late presence on the skin. The child was now sitting up in its cot, playing with its toys.

Case III.—Child of Mr. S., commission merchant, aged two years: Had been sick eight days. The feverish stage of incubation lasting four days, on the fifth day the eruption appeared. Had been out three days when seen. Eruption copious and confluent upon the face and chest; constitution not strong; child evidences signs of debility and depression, with possible sinking to be feared; pulse 100, feeble, but with a disproportionately high fever. Prescribed, as a stimulating diaphoretic, the following:

℞ Potass. chloras, ʒ j; liquor ammon. acet., ℥ j; spts. eth. nit., ʒ iv; aquæ ad. ℥ iv, s.g. A teaspoonful to be given every hour. At same time ordered the acid carbolic and sulphite of soda mixture (1 gr. acid carbol. to 10 soda sulphite) to be given every three hours.

As a topical application to destroy infectious nature or emanations from skin (there being other children in house), and to allay itching, the carbolate of glycerine was ordered ( ʒ ij acid carb. to glycerine ℥ iij) to be applied with a feather to the whole surface of the body, at least once a day, oftener if it should appear necessary. To quote the father's own words, "As soon as we began to use the remedy the fever abated, and the eruption began to wither and desicate, and in about six or seven days had entirely disappeared." No one contracted the disease from this case.

Cases IV. and V.—Mrs. M.'s children, aged respectively two years and nine months; of ordinary strength of constitution: Eruption had been out three days; fever slight; eruption not copious; case of a mild character; had both been vaccinated. Gave no diaphoretic mixture. Used only the carbolate of soda mixture internally every three hours, and the topical application of carbolate of glycerine (diluted for youngest child) upon the skin. These children both did well. The eruptions withered away and rapidly disappeared.

Case VI.—Mrs. L. McD., a married woman, aged about thirty, nursing an infant six months old: The mother contracted the disease, and the eruption made its appearance on fourth day, of a copious character, not confluent. Fever high; great thirst; and pains in back, head and bones, very distressing. Gave Seidlitz powders as aperient, and prescribed above remedies in following doses: Acid carb. grs. ij; sodæ sulphitis grs. xv., with glycerine, every three hours. The diaphoretic mixture also, during the first two days, or until fever abated, in liberal doses, and the usual topical application to the skin or eruption. In this case the effect of the treatment was most marked; the pustules immediately began to decline, not going on to fill or mature as is usual in small-pox; the fever subsided on the second day; the pains left simultaneously with the fever; the sleeplessness, which had been a distressing symptom before using the medicine, was succeeded by comfortable rest during the first night after taking medicine, and patient continued to rest well after. The eruption, which had begun to desicate on second day of treatment, began to scale off on the fourth day, and soon entirely disappeared, leaving a surface free from any traces of its former presence.

Strange to say, the child continued nursing throughout, and did not contract the disease. This woman is mother of six young children, none of whom contracted the disease.

Mr. F.'s children, two boys, aged respectively ten and four years, residents of St. David's Lane. Was called to see oldest child, who was first taken ill; had been ill for some days. Found head and face very much swollen; throat much inflamed; tonsils enlarged; eruption copious and confluent; body completely covered; fever high, and attended with constant delirium; eyes swollen and shut; and deafness present; child had been vaccinated in infancy.

Prescribed external applications of strong carbolate of glycerine to surface of body, and the following diaphoretic mixture:

℞. Potass. chloras, ʒj; liq. ammon. acet., ℥j; tr. aconit. rad., gtt. xxxij; spts. eth. nit., ʒiv; aquæ ad. ℥iv. A teaspoonful every four hours. Made use of a mouth-wash for fauces and tonsils of potass. chloras. Ordered carbolate of soda mixture every three hours.

This patient appeared to improve during the first four days, after which it refilled again, or rather an eruption succeeded it of what might be termed copious white blisters, filled with a thin milk serum or fluid. The delirium suddenly increased, the throat became much worse, and the patient refused all fluids, even medicine.

By this time the tonsils were extensively ulcerated. I prepared a lotion of carbolic acid, 1 to 100 of equal parts of glycerine and water. This enabled the patient, after a few hours' frequent application, to take some milk. Beef-tea was now ordered in spoonful doses every hour, with tart drinks. The patient continued insensible, and in a few hours afterwards showed signs of sinking, succeeded by a feeble pulse, coldness of surface, shiverings; and finally patient died on 14th day, in a state of collapse. My two important mistakes or omissions in this case seemed to me to have been omitting to immerse the whole body in a warm bath in the beginning of the case, or even later, which might have been daily repeated; and not using carbolic to the throat affection when first seen, and depending too much upon chlorate of potash. The throat difficulty seemed to be the pivot upon which the result of the case depended. Altogether, the case was the most severe I had seen for years, and had been contracted from a straw bed which had been thrown out of a neighboring house, upon which two patients, a mother and child, attended by a prominent medical gentleman, had died of the most severe and confluent type of the disease. The second boy came under my care during the fever stage, and I at once began the internal administration of the carbolic acid and sulphite of soda mixture, paying great attention to the skin and throat, to which I applied a very weak carbolic acid lotion. The bowels were kept relieved by castor oil; and the eruption, which appeared on the fourth day, began to decline on the sixth day, and was entirely gone on the eighth. This child had been tried with vaccine, but unsuccessfully. Wherever administered in early stage of the disease, the pustules have been prevented from maturing; and in no instance has any one contracted the disease from those thus treated.

This treatment is essentially the same as that followed by Dr. Boyer, of Philadelphia, and published in the *Medical and Surgical Reporter*. He gives a solution of 2 grs. carbolic acid, with 15 to 20 grs. sulphite of soda, every three hours, but with *no other treatment* than an ordinary purge during the initiative or forming fever.

The result of Dr. Boyer's experience with this plan of treatment, which seems to have been large, he gives as follows: "The result, after several months' trial, with myself and son, has been that, in *every case of variola* and confluent small-pox, on the fourth day of the eruption the swelling of the face abated, the pulse fell to a normal rate, the tongue commenced clearing, and the eruption began to dry up, and the pustules withered and shriveled. By the seventh and eighth day of the eruption the patient was convalescent, without a sign or mark of having had small-pox, after the slight desquamation of the light scales or scabs fell off. In no case by this treatment did the pustules positively mature, but always dried up before maturation. Externally, any soothing application for the first three days is all that is required to allay itching, etc.

The above extract from Dr. Boyer's statement of his experience fully accords with my own observations — which, however, have been necessarily somewhat limited — except in two particulars: first, that the eruption began in every case to wither on the second day after the remedy had been administered; and again, that no one, so far as I have been able to ascertain, contracted the disease from the patients under treatment. I am induced to report this plan of treatment, with my views as to the philosophy of it, in the hope that others who may have opportunity may be induced to take it up and give it a more wide-spread and extended trial than it has yet received.—*Canada Medical Record.*

